# Mesenchymal Stem Cells (MSCs) as a Potential Therapeutic Strategy in COVID-19 Patients: Literature Research

**DOI:** 10.3389/fcell.2020.602647

**Published:** 2020-11-19

**Authors:** André Coelho, Rui Damásio Alvites, Mariana Vieira Branquinho, Susana G. Guerreiro, Ana Colette Maurício

**Affiliations:** ^1^Biotecnologia Medicinal, Escola Superior de Saúde do Porto, Instituto Politécnico do Porto, Porto, Portugal; ^2^Departamento de Clínicas Veterinárias, Instituto de Ciências Biomédicas de Abel Salazar, Universidade do Porto, Porto, Portugal; ^3^Centro de Estudos de Ciência Animal, Instituto de Ciências, Tecnologias e Agroambiente da Universidade do Porto, Porto, Portugal; ^4^Departamento de Biomedicina, Faculdade de Medicina, Universidade do Porto, Porto, Portugal; ^5^Instituto de Investigação e Inovação em Saúde (I3S), Universidade do Porto, Porto, Portugal; ^6^Instituto de Patologia e Imunologia Molecular da Universidade do Porto, Porto, Portugal

**Keywords:** cell-based therapies, secretome, mesenchymal stem cells, coronavirus, SARS-CoV-2

## Abstract

In 2019, an outbreak of an unknown coronavirus – SARS-CoV-2 – responsible for COVID-19 disease, was first reported in China, and evolved into a pandemic of huge dimensions and raised serious concerns for global health. The number of critical cases continues to increase dramatically, while vaccines and specific treatments are not yet available. There are several strategies currently being studied for the treatment of adverse symptoms of COVID-19, that encompass Acute Lung Injury (ALI)/Acute Respiratory Distress Syndrome (ARDS), extensive pulmonary inflammation, cytokine storm, and pulmonary edema, due to virus-induced pneumonia. Mesenchymal stem cells (MSCs) are at the origin of new revolutionary treatments, which may come to be applied in such as Regenerative Medicine, Immunotherapy, Tissue Engineering, and Cell and Molecular Biology due to immunomodulation and anti-inflammatory activity. MSCs have already been studied with positive outcomes for other lung pathologies, thus representing and being identified as an important opportunity for the treatment of COVID-19. It has recently been shown that these cells allow hopeful and effective therapies for serious or critical COVID-19, minimizing its adverse symptoms. In this study we will analyze the MSCs, their origin, differentiation, and therapeutic potential, making a bridge with the COVID-19 disease and its characteristics, as a potential therapeutic strategy but also reporting recent studies where these cell-based therapies were used for the treatment of COVID-19 patients.

## Introduction

Since the end of 2019 that a series of unexplained cases of pneumonia caused by an unknown virus were reported in Wuhan, China. The research began and on 12 January 2020, when the World Health Organization (WHO) identified the newly discovered virus as the “2019 Novel Coronavirus” (2019-nCoV), formally naming the infection as Coronavirus Disease 2019 – COVID-19 – on 30 January. On the same day, the International Committee on Taxonomy of Viruses identified the 2019-nCoV as a Severe Acute Respiratory Syndrome 2 – SARS-CoV-2 (Sun et al., 2020b). First outbreak reported in China, this disease quickly spread to all continents mainly due to international flights, becoming a pandemic recognize by the WHO since January 2020, affecting nowadays 213 countries and territories, with more than 11 million active cases and more than half a million deaths, representing a serious global public health emergency (Table 1) (Worldometer, 2020).

**TABLE 1:** Total confirmed cases, total deaths, and total cases per 1M population of the 10 countries most affected by COVID-19, and the data from Portugal for comparison (Data consulted on October 10 at worldometers.info/coronavirus) (Worldometer, 2020).

**Country**	**Total Cases**	**Total Deaths**	**Total cases/1M pop**
United States	7,895,738	218,685	23,816
India	6,979,423	107,450	5,044
Brazil	5,057,190	149,692	23,746
Russia	1,285,084	22,454	8,805
Colombia	894,300	27,495	17,525
Spain	890,367	32,929	19,041
Argentina	871,468	23,225	19,234
Peru	843,355	33,158	25,482
Mexico	809,751	83,507	6,262
France	691,977	32,583	11,567
Portugal (49th)	83,928	2,062	8,238

SARS-CoV-2 is characterized as a coronavirus, belonging to the cluster of β−coronavirus. COVID-19 appears as the third zoonotic disease caused by this type of virus, after Severe Acute Respiratory Syndrome (SARS) and the Middle East Respiratory Syndrome (MERS). The first cases identified as COVID-19 were related to the Huanan seafood market, since environmental samples were collected from the seafood market, and consequently, it is believed that SARS-CoV-2 mutated and an intermediate host that infected the patient zero in the market allowed the interspecies transmission to humans (Li et al., 2020b). Although it has not been possible to determine with certainty whether a particular specie carries the SARS-CoV-2, studies demonstrated that SARS−CoV−2 was a chimeric virus that originated from a bat coronavirus and another one of undetermined origin. As a well-known natural reservoir for α-coronavirus and β-coronavirus, bats are carriers of distinct SARS-like-CoVs (Sun et al., 2020a). Therefore, various researches reinforced the hypothesis that the transmission chain originated from animals (bats) to humans.

### Structure and Mechanism of SARS-CoV-2

Coronaviruses, or Orthocoronavirinae, are enveloped, positive, single−stranded large RNA viruses that researchers previously thought that transmitted disease only to animals until the world witnessed the SARS epidemic outbreak caused by SARS-CoV in 2002. Currently are known 7 CoVs (SARS-CoV-2 included) responsible for causing human respiratory illnesses, although only SARS-CoV-2, MERS-CoV, and SARS-CoV have been associated with outbreaks with high infection rate and high mortality (Li et al., 2020a). Coronavirus dimensions can vary from 26 to 32 kb in length, 60–140 nm in diameter, with peptameric spiky projections on its external surface, looking as crown like spikes under the electron microscopy (Madabhavi et al., 2020). The spike protein is mainly responsible for the occurrence of cell tropism and inter-species transmission, since it allows the virus to bind to a cell receptor and its subsequent penetration into the cell through its membrane. Researchers discovered that angiotensin−converting enzyme 2 (ACE-2) is the receptor for SARS−CoV−2 (Madabhavi et al., 2020; Sun et al., 2020a) in healthy human lung, being expressed on alveolar epithelial cells type I and II. The attachment of SARS−CoV−2 to ACE-2 leads to an elevation of the expression of ACE-2, that can trigger injures on alveolar cells. Sequentially, these lesions can promote severe systemic reactions and culminate in death. ACE-2 is expressed in other tissues such as the kidney, liver, heart and other organs, this expression being the reason behind critical patients suffering not only from respiratory complications, but multiple organ dysfunction as well (Sun et al., 2020b). The nasal epithelium was found the first site of infection but also in transmission among patients, with symptomatic but also asymptomatic clinical spectrum (Xiao et al., 2020). Like it is observed in other SARS-CoV infections, the S protein of SARS-CoV-2 is able to bind the ACE-2 which allows the cell entering catalyzed by transmembrane protease serine-2 (TMPRSS2) protease mostly in type II alveolar cells in the pulmonary parenchyma but also other target cells that express ACE-2 (Spike, 2010).

### Epidemiological Characteristics of COVID-19

As of October 6, 2020, the total number of confirmed cases was 35,736,102, including 1,046,546 deaths in all five continents, and a total of 26,894,740 recovered patients (Worldometer, 2020), nowadays a second vague of infection is being observed worldwide (Table 1). All ages are vulnerable to this lethal virus infection, and its transmission is made in the course of coughing and sneezing, through infected droplets by infected patients, able to spread about 1–2 m and deposit on surfaces. One of the main factors of the high rate of transmission of this virus is the fact that can also occur from asymptomatic people before the start of symptoms. The incubation period varies from 2 to 14 days. The first studies about COVID-19 transmission profile revealed that the basic reproductive number (R0) of 2019-nCoV is positioned between 1.4 and 3.9 (Bulut and Kato, 2020). Researchers also noticed a 3.25:1 ratio of male to female deaths, a fact probably related to the higher numbers of ACE-2 receptors present in male alveoli. The age range of deaths by COVID-19 was 70 years or older, although the deaths of younger age groups are now increasing. Findings made so far indicate that the progression of the disease is faster in older individuals, especially on those who have comorbidities, than in the young (Sun et al., 2020b).

COVID-19 can be clinically divided in five groups: asymptomatic, mild, moderate, severe, and critical. The symptoms of these categories vary, with asymptomatic having no symptoms with a positive SARS-CoV-2 PCR test. The mild group manifests itself through signs such as fever, myalgia, cough, fatigue, sore throat, and sneezing, runny nose without evolution to pneumonia, basically signs of acute upper respiratory tract infection. More aggressive symptoms such as fever, cough and pneumonia with no indication of shortness of breath or hypoxemia should be included in the moderate group. The severe group includes cases of rapid evolution (in most cases in 1 week), where oxygen saturation below 92% promotes signs of central cyanosis, dyspnea and other symptoms of hypoxemia. Finally, the critical patients present acute ARDS or respiratory failure with multiple organ dysfunction, requiring mechanical ventilation and life support (Bulut and Kato, 2020). The main pathologic traits for COVID-19 patients in the severe or critical groups include the development of extensive pulmonary edema, pulmonary inflammation, diffuse alveolar damage with cellular fibromyxoid exudates, hyaline membrane formation and hypoxemia. Even though the pathologic alterations are similar to ALI/ARDS, also witnessed in MERS and SARS coronavirus infections, more serious phenomena of inflammatory cytokine storm, severe inflammatory exudation, milder pulmonary fibrosis and consolidation, pulmonary edema and serious inflammatory exudation were observed in severe or critical COVID-19 (Liu et al., 2020). The severe and critical patients will have long-term health impact of COVID-19. Taken all together, MSCs-based therapy seems to be a possible strategy for patients’ treatment and rehabilitation, mostly what concerns pulmonary parenchyma regeneration.

### Prevention and Treatment of COVID-19

At the date of October 2020, there is not yet a vaccine against COVID-19 available. The vaccine is considered as an effective prophylactic strategy for control and prevention, and is being researched and developed in about 90 institutions worldwide. The knowledge of the structure of the SARS-COV-2 spike protein (S) should enable rapid evaluation and further studies to optimize vaccination strategies. Several possible treatments are also being studied, some of them were already considered ineffective for COVID-19, such as chloroquine and hydroxychloroquine. The multiple treatment strategies include antiviral agents like Remdesivir designed for Ebola treatment, drugs routinely used to treat autoimmune diseases and malaria which drew attention for their potential as broad-spectrum antiviral drugs; corticosteroids to suppress lung inflammation; several immunotherapies; convalescent plasmas transfusion. Finally, new therapies like blocking agents that present affinity to bind to ACE2 receptor and the use of cell-based approaches such as MSCs, have been studied and tested as potential approaches (Zhai et al., 2020).

The currently available treatment to COVID-19 depends basically on the patients’ immune system. COVID-19 can trigger a destroying immune overreaction, contributing to the worsening of the disease. When the immune system is activated in a reactive way, the physiological mechanisms of containment of the virus trigger the production and release of several inflammatory mediators, resulting in a severe cascade of cytokines. This mechanism can culminate in injuries to several organs, followed by acute cardiac injury, edema, ARDS, changes in gas exchange, secondary opportunistic infections, multi-organ failure and death. That said, preventing or attenuating the development of the cytokine cascade seems to be the way forward in the treatment of COVID-19 infected patients (Atluri et al., 2020). On the other hand, until an effective treatment is established, the management of the pandemic essentially involves the control of infections and the community transmission of the virus. The set of strategies should include ensuring early diagnosis with effective data sharing, isolation of the infected, maintenance of social orders, quarantine, the fidelity of disseminated information, and supportive treatments. For individuals, protective measures like the improvement of personal hygiene measures, the use of protective barriers such as masks, visors and gloves and the guarantee of good ventilation in closed spaces, can effectively reduce the transmission of the virus (Madabhavi et al., 2020). In summary, COVID-19 is an unprecedented challenge for clinicians because of the high transmission rate (Sun et al., 2020a), making the number of cases classified as severe increasing markedly worldwide while effective and precise treatments are still absent. Efforts are being made in a global perspective to find solutions or treatments (Zhai et al., 2020).

Cell-based therapies, particularly those involving MSCs or their extracellular vesicles (EVs) and secretome, have recently started to be used in the treatment of COVID-19 respiratory disease (Khoury et al., 2020). MSCs are non-hematopoietic, multipotent cells, able to differentiate into the three dermal lineages, and are present in tissues of different sources, ranging from fetal to many adult tissues. Due to their proven benefits when used in Regenerative Medicine, MSCs are good options for use as cell-based therapies in regenerating and replacing injured tissues and organs, but their potential goes way beyond that, with multiple therapeutic utilities and positive outcomes with immunomodulatory and anti-inflammatory properties in neurodegenerative, autoimmune, and cardiovascular diseases (Ullah et al., 2015; Li and Hua, 2017; Denton et al., 2018; Shammaa et al., 2020). While lodged in the lungs, the MSCs can balance the inflammatory response and release mediators including antimicrobial peptides, angiogenic growth factors, and EVs (Khoury et al., 2020), presenting MSCs as an valuable option to be used to treat COVID-19. As a matter of fact, MSCs present immunomodulatory capacity, so, it is observed their ability to prevent and attenuate the cytokine storm, reducing the morbidity and mortality. This literature review aims at discussing MSCs as a strategy for the medical approach of severe or critical COVID-19 patients and further understanding the impact of MSCs origin and *in vitro* culture conditioning methods, as well as their characterization, on their therapeutic potential and clinical significance toward COVID-19 disease. Recent clinical trials outcomes, with use of MSCs for COVID-19 will be analyzed. It seems that expanded umbilical cord mesenchymal stem cells (UC-MSCs) may be the most efficient possible treatment available, associated to supportive therapies, for severe clinical cases (Atluri et al., 2020; Xiao et al., 2020).

## Origins, Development and Clinical Applications of Mesenchymal Stem Cells (MSCs)

### Origins, Discovery and Brief Historical Context of MSCs

Friedenstein was one of the pioneers in the study of stem cells and the identification of multipotential stromal precursor cells. Based on his 1960s and 1970s discoveries, it was shown that mouse bone marrow (BM), like other blood-forming organs, contained clonogenic progenitor cells that, once in culture, could originate fibroblasts and other mesodermal cells. He also understood that these cells were precursors of cartilage and bone-forming cells but did not belong to the hematopoietic cell lineage (Frieedenstein et al., 1968). From BM, Friedenstein was also able to isolate adherent cells identical to fibroblasts that grew rapidly *in vitro* and were able to form clonogenic colonies called colony forming unit-fibroblast [CFU-F]. Cells isolated from CFU-F had the additional ability to differentiate into chondrocytes, osteocytes, osteoblasts and adipocytes *in vitro* (Friedenstein et al., 1974). Arnold Caplan later called these cells “Mesenchymal Stem Cells,” establishing similarities with stem cells originating from mesodermal tissues at the embryonic level, having been able to promote their growth from adult tissues (Caplan, 2009). Haynesworth et al. (1992b) was able to grow and expand BM-MSCs from the iliac crest’s BM collected in human donors, revealing the human BM’s ability to produce cells with the potential to grow in culture and with osteogenic capacity. SH-2 and SH-3 were identified as specific cell surface antigens on MSCs by the same group (Haynesworth et al., 1992a). Subsequently, Barry et al. (2001) established CD105 and CD73 as ligands corresponding to SH-2 and SH-3.

**FIGURE 1:**
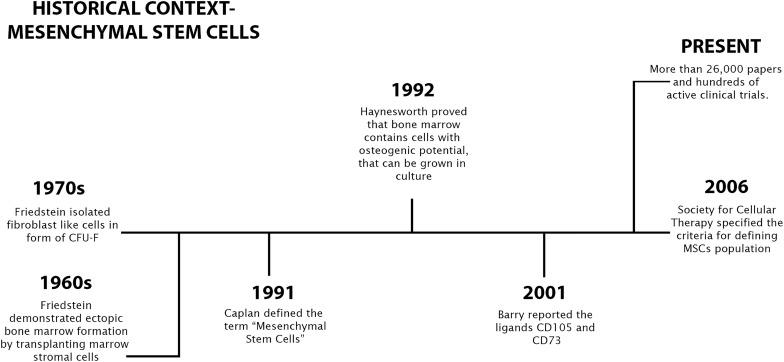
A brief history of mesenchymal stem cells discovery and research.

In the next years, massive interest in MSCs studies was established and, even though BM was exploited as the source organ of MSCs in the first place, quickly adult adipose tissue (AT) was established as a main niche of MSCs, demonstrating an equally interesting multipotency *ex vivo* (Gomez-Salazar et al., 2020). The rapid interest that fell on MSCs, associated with their demonstrated clinical relevance and some controversy associated with the characteristics and nomenclature attributed, raised the need to establish an official and more restricted definition for these cells. The International Society for Cellular Therapy (ISCT) in 2006 (Dominici et al., 2006), established the parameters that were established as the four minimum criteria that should be used to define cells as MSCs, which were quickly accepted by the scientific community: (i) be plastic adherent; (ii) expression of CD105, CD90, and CD73 cell surface antigens; (iii) no expression of CD45, CD19, CD14 CD11b, CD34, CD79a, and HLA-DR cell surface antigens; (vi) Capacity to differentiate into adipocytes, chondrocytes and osteoblasts. MSCs have been frequently re-baptized with various names such as “Mesenchymal Progenitor Cells,” “Multipotent Adult Progenitor Cells” or “Multipotent Adult Stem Cells” that diverged only vaguely from the original definition. Other denomination such as “Multipotential Stromal Cells” or “Mesenchymal Stromal Cells,” even fitting in the MSCs acronym, are based on radical differences in terms of biologic meaning (Gomez-Salazar et al., 2020). It is true that MSCs share several characteristics with stem cells, but some of their elements related to permanent cell lineage repletion *in vivo* do not allow them to be considered as “true stem cells” and require the use of a different denomination. More recently, one work has established MSCs as natural precursors for adventitial cells, perivascular fibroblasts and pericytes, pointing to a stromal origin for MSCs (Crisan et al., 2008). Although more recently the 2006 ISCT guidelines have advised the use of the term “Multipotent Mesenchymal Stromal Cells,” the original term “Mesenchymal Stem Cells” continues to be mostly used. Finally, to highlight the fact that the therapeutic action of these cells may be primarily related with releasing growth factors and cytokines, Arnold Caplan supported the use of the term “Medicinal Signaling Cells” (Caplan, 2017). To simplify, to make everything uniform, and facilitate bibliographic research, the term “Mesenchymal Stem Cell” remains the most commonly used name. Recent findings demonstrate that MSCs can be isolated from any mesenchymal tissue or niche that manifests regenerative capacity, and that these cells have basic Stem Cell capabilities such as clonogenic potential, self-renewal, and ability to regenerate tissues *in vivo* and multi-lineage differentiation capacity. Some MSCs that are cultured *in vitro* lack specific identifying markers (Lanza et al., 2009), so the variable expression level of these markers could probably arise from differences between species, tissue sources, and culture conditions that we will discuss in this review. MSCs represent an important tool in the development of new possible therapies with applications in regenerative medicine, immunotherapy, tissue engineering, and cell and molecular biology with almost 10,000 papers published in the last 40 years [search, in the PUBMED.gov search engine, by the term *“(Mesenchymal stem cell) OR (MSCs)*,” on 08/31/2020] and hundreds of clinical trials made over the years, with more than 500 active trials currently (Figure 1) (research, in the ClinicalTrials.gov database, under the terms “*MSCs*,” on 08/31/2020).

### Sources, Isolation, and Cultivation of MSCs

**FIGURE 2:**
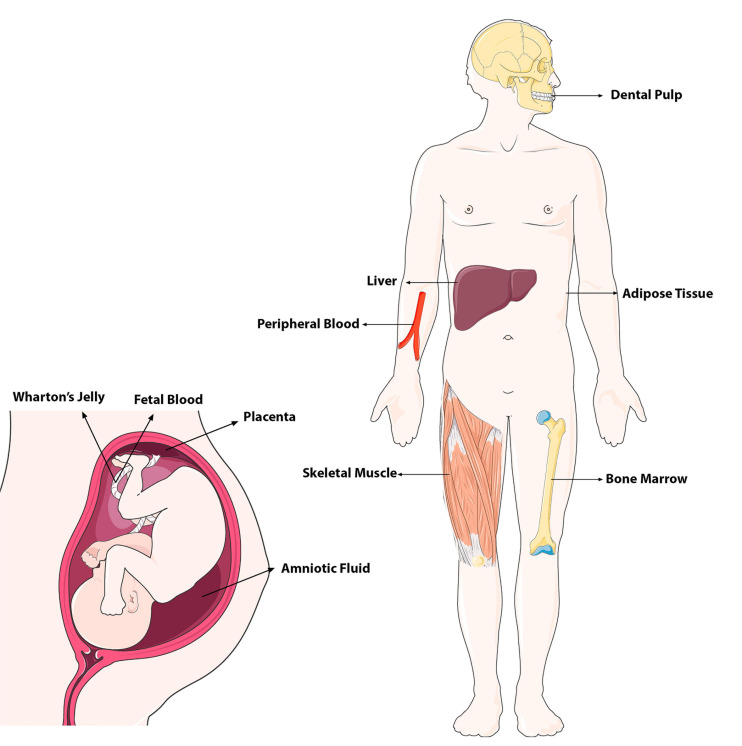
Adult and fetal tissue sources (niches) of MSCs in the body (modified from Servier Medical Art, licensed under a Creative Common Attribution 3.0 Generic Licence. https://smart.servier.com/).

Stem cells can be categorized considering their profile of differentiation and source within the human body. Discovered originally in the BM, this type of cells is still recognized as the more promising for clinical application and research, although some alternative sources including AT, birth-derived tissues, amniotic fluid (AF) and placenta, dental pulp (DP), olfactory tissues, synovium and synovial fluid, endometrium, muscle, and peripheral blood — have since been identified, as we can see in Figure 2 (Berebichez-Fridman and Montero-Olvera, 2018).

In theory, MSCs can be isolated from almost any tissue in the human body. Despite this, it is necessary to consider some practical limitations, such as the associated difficulty, the degree of invasion of the collection method and the characteristics of the donor himself. Parameters such as tissue source, isolation technique and the culture medium used can alter the properties of the collected human MSCs. MSCs can differentiate in the neuroectodermic and endoderm lines, in addition to the already known capacity for mesodermal lineage differentiation (Mushahary et al., 2018). The developmental origin of mesenchymal tissues, including not only mesoderm but also the cranial neural crest, may be the explanation for this ability. It is important to note that recently it was demonstrated that MSCs are mostly derived from the neural crest and neuroepithelium, even though traditionally it was considered the mesodermal origin (Uccelli et al., 2008). Embryonic Stem Cells (ESCs) are totipotent because they can form both embryonic and extraembryonic structures. Moreover, ESCs can proliferate indefinitely under specific culture conditions and retain the capacity to differentiate into cell types from the three embryonic germ layers. On the other hand, adult stem cells are undifferentiated multipotent stem cells obtained from adult individuals and differentiate into the cell types that constitute their respective source tissue (Uccelli et al., 2008; Berebichez-Fridman and Montero-Olvera, 2018).

#### Bone Marrow

The procedure to obtain BM-MSCs is a highly invasive and can result in pain or infection, making the selection of other sources, like peripheral blood or surgical remnants such as AT, preferable (Berebichez-Fridman et al., 2017). The cell yield, longevity, and potential for differentiation decrease with donor age (Choudhery et al., 2014). About 0.01 and 0.001% of the total nucleated cells isolated from BM aspirates are MSCs (Salem and Thiemermann, 2010). Besides that, these cells continue to be widely used, as they can be effortlessly isolated from a small quantities of aspirate and have a short culture time, being able to double 40 times its numbers in about 8–10 weeks of culture (Salem and Thiemermann, 2010). In terms of differentiation, BM-MSCs have an advantage when compared to other sources, with high multi-lineage differentiation and proliferation capacity. This type of stem cell is the most widely investigated, with active studies in arterial hypertension, ischemic heart failure, digestive system, treatment of spinal cord injuries, among others, and therefore is considered to be the gold standard (Choudhery et al., 2014).

#### Adipose Tissue

Adipose tissue is the second most reputable source of MSCs, with an estimated 98–100% cell viability. The morphological, phenotypical, and functional characteristics of AT-MSCs are similar to BM-MSCs (Choudhery et al., 2014). Besides their stability in long-term cell cultures, AT-MSCs can expand effectively *in vitro* and possess high multi-lineage differentiation potential. AT is a more advantageous autologous niche of MSCs for tissue engineering than BM (Choudhery et al., 2014). Its main advantage as a MSCs source is convenience, as human AT is usually abundant throughout the body and is easily accessible through liposuction procedures.

#### Birth-Derived Tissues

Currently, umbilical cord blood (UCB) is accepted as a source of hematopoietic stem cells, but also of MSCs and the abundance of UCB, availability of donors, facility of acquisition, and reliability of sample collection are relevant advantages (Berebichez-Fridman and Montero-Olvera, 2018). UCB-MSCs are more immature than other types of adult stem cells, and do not evoke a strong immune rejection response in an allogeneic recipient. UCB-MSCs can be cryopreserved in vast quantities for later cultivation and research, but also for the treatment of hematologic pathologies, and possess osteogenic, chondrogenic, adipogenic, and myogenic differentiation potential. Lately, Wharton’s Jelly (WJ) has gained attention as an excellent source of MSCs, as these MSCs present immuno-privileged and immunomodulatory phenotype (Caseiro et al., 2015).

#### Amniotic Fluid and Placenta

The phenotype of cultured cells collected from AF is similar to that of BM-MSCs. Cultured cells obtained from this source can differentiate into mesenchymal lineages, with a high self-renewal capacity and a doubling time of 36 h, maintaining a normal karyotype even at late passages. Placenta-derived MSCs also express embryonic stem cell markers and can differentiate into mesenchymal as well as hepatic, pancreatic, and neuronal lineages (Spitzhorn et al., 2017).

#### Mobilized Peripheral Blood

Peripheral blood stem cells may be mobilized from healthy donors using granulocyte-macrophage colony-stimulating factor (GM-CSF). The underlying mechanism involves several adhesion molecules that facilitate the binding of stem cells to BM and their disruption allows for their release into the circulation. A large volume of blood and, therefore, a higher quantity of MSCs can be collected compared to BM-MSCs (Pouryazdanpanah et al., 2018).

#### Synovium and Synovial Fluid

In humans, cartilage and synovium originate from a common pool of cells during synovial joint development. Synovia-derived MSCs are far superior to cells derived from the skeletal muscle and AT, as determined by their *in vitro* expandability, differentiation potential, and epitope profiles (Berebichez-Fridman and Montero-Olvera, 2018). Their superior chondrogenic and proliferation potential has been reported by several groups, making these cells fair candidates for cell therapy applications, particularly regarding osteoarticular disorders (Branquinho et al., 2012).

#### Dental Pulp

DP-derived MSCs specialize in odontoblasts, which produce dentin. These cells are obtained from pulp tissue and the periodontal ligament, and are extracted by an enzymatic digestive process or explant method. DP-MSCs are easy to cryopreserve and, similarly to AT-MSCs, possess immunomodulatory properties. In addition to differentiating into osteo/chondroblasts and adipocytes, DP-MSCs can also differentiate into neuronal lineages (Caseiro et al., 2019).

#### Olfactory Tissues

The *lamina propria* of the olfactory mucosa is the niche for olfactory mucosa mesenchymal stem/stromal cells (OM-MSCs) that are derived from the neural crest. Different works involving OM-MSCs revealed its fibroblastic-like cytomorphology, development of low density colonies, the plastic-adhesion, the expression of expected surface markers and also the capacity for differentiation, not only the classic tri-differentiation but also other pathways such as neuroglial and myogenic (Alvites et al., 2020a). Its secretome has also started to be analyzed, and it was possible to identify several bioactive molecules with pro-regenerative and immunomodulatory functions (Ge et al., 2016). The clinical use of these cells can be advantageous due to their anatomical location, easily accessible, and also to their wide distribution in the nasal cavity. Additionally, previous studies have already shown that OM-MSCs can be maintained in culture for extended periods without chromosomal or tumorigenic changes being observed. This capacity, affected by the donor’s age, seems to be related with some inhibiting of apoptotic activity and conservation of telomeric activity (Alvites et al., 2020a). OM-MSCs have already been isolated from the olfactory mucosa of several species (Veron et al., 2018), including humans (Delorme et al., 2010), and their therapeutic effectiveness has also been tested with promising results in neurodegenerative diseases of the central nervous system, peripheral nerve injuries, autoimmune diseases and even myocardial tissue injuries (Alvites et al., 2020b).

#### Isolation and Cultivation

Mesenchymal stem cells are plastic-adherent cell populations, isolated through procedures involving tissue mincing, enzymatic digestion, and cell growth on a plastic surface. The two main procedures are the enzymatic and explant protocols (Mushahary et al., 2018). In the explant method, one of the earliest techniques applied, the tissue is rinsed with a saline solution and mechanically split into smaller fragments. Fragments are further placed on a culture dish and covered in adequate culture medium. After some days in culture, MSCs start outgrowing from the explants’ limits, and as they reach total confluence, explants can be carefully detached. The enzymatic method entails a more complex protocol. After rinsing with a saline solution and mechanical splitting into smaller fragments, identically to the first steps on the explant method, the tissue is incubated with an enzymatic solution. As a result, singe cells or aggregates are released and remain suspended in the enzymatic solution. After centrifugation for elimination of the enzymatic solution, the cell pellet is re-suspended in adequate culture medium and seeded in culture dishes. MSCs therapeutic potential toward a specific tissue regeneration lies not only on the MSCs tissue of origin, but also on their potential *itself* and on their secretion potential of bio-factors with pharmacological properties, the so called secretome. MSCs protocols between groups regarding isolation, culture conditions and characterization varies immensely, impairing the reproductivity of results between different research groups. A critical parameter when considering MSCs is the cell potential toward regeneration. It has been reported to be influenced by cell passage number/age, and cryopreservation/defrosting cycle-related injury (Rendra et al., 2020). An improved therapeutic efficacy of MSCs is greatly influenced by optimized culture condition protocols (Alvites et al., 2020a).

### Characterization and Phenotype of MSCs

As previously mentioned, in 2006 the ISCT proposed four minimum criteria to characterize cells as MSCs for research purposes (Dominici et al., 2006): be plastic-adherent in standard culture conditions (Dominici et al., 2006). Second, the MSC population must express CD105 (known as endoglin, first known as SH-2), CD73 (known as ecto 5’ nucleotidase, first identified as SH-3), and CD90 (also known as Thy-1), and lack expression of CD45 (pan-leukocyte marker), CD34 (hematopoietic progenitors and endothelial cells marker), CD14 or CD11b (expressed on monocytes and macrophages), CD79a or CD19 (markers of B cells) and HLA class II. Lastly, MSCs most be able to differentiate into three different lineages: osteoblasts, adipocytes and chondroblasts. It’s crucial to know that these identifying criteria should not be confused by no means and does not represent adequate criteria and characteristics for MSCs used therapeutically and in clinical studies because the proposal is meant only to describe identifying criteria for research purposes. The homogeneity of these characteristics is relative as different MSCs population express different surface markers, an understandable feature, as MSCs present plasticity, a dynamic capacity to adapt and vary over time to different conditions. Different antigen sets are applied from different research groups, to characterize these cells, with different outcomes, thus, no specific marker was identified as to uniquely characterize MSCs. MSCs can differentiate into different cell types when an array of differentiation factors is used to mimic osteogenic, chondrogenic, or adipogenic *in vitro* microenvironment, for instance. Standardized procedures for culture conditions and MSCs characterization still remains a major challenge. Although the official criteria speak of tri-differentiation, it has long been known that MSCs can follow multiple types of differentiation and that is also a factor in the discussion of these criteria, with some studies suggesting the addition of other criteria factors for MSCs characterization. Some research groups suggest that to profile MSCs we must observe key parameters like CFU-F efficiency, MSC isolation, multilineage differentiation, immunomodulation, telomere length, trophic factor quantification, cumulative growth, and surface phenotype (Samsonraj et al., 2017). However, the ISCT guidelines are still the recommended criteria for the isolation and characterization of MSCs and are precise enough to combine the majority of the to-day existing knowledge, recognizing that future research, along with scientific breakthroughs, will probably lead to optimization of the criteria (Greif et al., 2020; Kurenkova et al., 2020).

### Multipotency Analysis of MSCs

**FIGURE 3:**
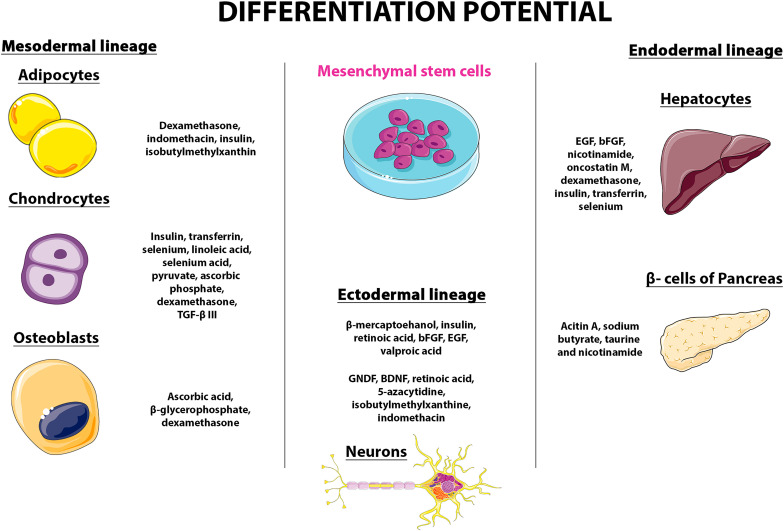
Differentiation potential and the mesengenic process (modified from Servier Medical Art, licensed under a Creative Common Attribution 3.0 Generic Licence. https://smart.servier.com/).

Considering MSCs are an interesting source for autologous transplantation and analysis of their cellular and molecular pathway has been performed, as well as the niche changes, as to assess on the cytokines, chemokines and other bioactive factors role in the differentiation potential of these cells (Alvites et al., 2020b). MSCs present a great differentiation potential toward mesenchymal lineages. Under specific *in vitro* inducing conditions, they can differentiate into adipogenic, osteogenic, or chondrogenic lineage. Differentiation can be induced *in vitro*, when seeding MSCs in specific differentiation supplemented media, as shown in Figure 3.

Regarding differentiation into the adipogenic lineage, supplementation includes dexamethasone, indomethacin, insulin, and isobutylmethylxanthine. As for the chondrogenic lineage, the cells can be cultured in Dulbecco’s modified Eagle’s medium supplemented with insulin, transferrin, selenium, linoleic acid, selenium acid, pyruvate, ascorbic phosphate, dexamethasone, and TGF-β III, and optionally IGF-1 and bone morphogenetic proteins-2. The osteogeneic lineage differentiation includes supplementation with of ascorbic acid, β-glycerophosphate, and dexamethasone (Mushahary et al., 2018). The differentiation into these three lineages is evaluated as for the presence of fat droplets (adipogenesis), proteoglycans and type II collagen synthesis (chondrogenesis), or mineralization of calcium deposits and increase in alkaline phosphatase expression (osteogenesis) (Sami et al., 2016). MSCs can differentiate into other lineages, such as the neurogenic, myogenic, tenogenic, and others as it was previously reported by several research groups (Branquinho et al., 2012; Ge et al., 2016). For instance, a process defined as *mesengenesis* has been reported by Caplan (1994), where MSCs originated myoblasts, ligaments, tendons and other cell types (Sami et al., 2016). Ever since, MSCs have been reported to originate cells from the heart, peripheral blood, cord blood, muscle, AT, lung, trabecular bone, intestine, kidney, liver, pancreas, synovium, skin, and even in the brain (Mushahary et al., 2018), being described as multipotent cells that representing a promising source for Regenerative Medicine.

### Clinical Significance of MSCs Therapy

The discovery of MSCs and their properties has increased the interest in deepening knowledge over their therapeutic features. MSCs are an excellent candidate for cell therapy since they are easily accessible – tissue collection and isolation are relatively simple, and proliferate *in vitro*. MSCs are easily preserved, maintaining their potential, and clinical trials have been showing promising results (Andrzejewska et al., 2019). Firstly studied because of their potential in Regenerative Medicine, other mechanisms of action have been studied and investigated in different areas like immunomodulation and inflammatory process, as well as the paracrine effects of MSC secretome (Caseiro et al., 2019). The attention is now focused on the secretome and EVs as candidates to explain the therapeutic effects of MSCs (An et al., 2020; d’Angelo et al., 2020; Varderidou-Minasian and Lorenowicz, 2020).

Mesenchymal stem cells therapeutic potential toward restauration of tissue function is associated with three different, but complementary, mechanisms. “Homing” is the term used to describe their aptitude to migrate toward the lesion site, due to chemotactic mechanisms, mediated by integrins and selectins. The second mechanisms, as described before, is the multi-lineage differentiation capacity, promoting cell engraftment and tissue regeneration (Caplan and Dennis, 2006). The third mechanism entails the secretion of bioactive factors, modulating both local and systemic physiological processes (Biju and Jack, 2013). In animal models, there are several registered successful clinical trials with promising results in several different tissues like myocardial, renal, hepatic, neuronal, among others (Shammaa et al., 2020). Therefore, the regenerative properties of MSCs are the main feature studied, with a focus on degenerative conditions that are widely exploited. The most recent approaches in regenerative medicine employ *scaffolds* (Pedrosa et al., 2017; Vizoso et al., 2017) or MSC engineering on both genetic and architectural levels (Shammaa et al., 2020).

Apart from their tissue regeneration potential, MSCs have been described as modulators of the immune system, attenuating tissue impairment due to inflammatory processes. The modulation of the immune system by the MSCs is mediated by the secretion of soluble factors and direct contact with immune cells. Studies have reported MSCs interaction and suppression of immune cells, native and adaptive (Alvites et al., 2020a). MSCs also induce regulatory T cells and suppress self-reactive T-effector responses (Pedrosa et al., 2017). The ability to modulate the immune system by cell contact has been investigated, and it is specially of benefit when considering autoimmune conditions and neurodegenerative disorders (Shammaa et al., 2020).

### Secretome and Conditioned Media as a New Therapeutic Strategy

The secretome is defined as substances, factors/molecules secreted by the cells to the extracellular matrix, a promising mechanism considering regenerative cell based-therapies and cellular mechanisms. The secretome includes soluble proteins, free nuclei acids, lipids, EVs among others. The use of cell-free therapies, using, for example, the conditioned media as a source of MSCs’ secretome, reveals several benefits such as safety, once that the application of the secretome resolves potential impairments as immune compatibility, tumorigenicity, emboli formation and the transmission of infections. It has other advantages like the storage without potentially toxic cryoprotectors, it is more economic and the time of expansion and maintenance can be considerably reduced. MSCs’ secretome has diverse mechanisms and great potential in immunomodulation, anti-apoptotic activity, wound healing and tissue repair, neuroprotective effect, angiogenesis regulation, antimicrobial effect, and antitumor effect (Caseiro et al., 2019; Alvites et al., 2020a). EVs, elements of the MSCs secretome, deserve a specific mention due to the recently accelerated studies of its clinical applications. EVs are exosomes, microvesicles and apoptotic bodies, that transport biologically active molecules and genetic information to target cells, thus affecting their potential and function (Trohatou and Roubelakis, 2017).

## MSCs as a Potential Therapy for COVID-19

According to the WHO, the control of COVID-19 has mainly directed on infection prevention, case detection, and monitoring, since the currently approved treatments are of support and not directly envisioning the cure of the pathology. Although vaccines are being developed in order to reduce the infection rate of COVID-19, a developing demand to cure the increasing number of patients who develop pneumonia and other critical symptoms is emerging. That’s where MSCs can have a crucial role, by minimizing the symptoms of COVID-19 and giving a chance to the patient immune system to act against the virus and promote the pulmonary parenchyma regeneration (WHO, 2020).

As previously reported, the most clinical used MSCs are the ones obtained from the autologous BM. The other MSCs available for clinical application are the ones from the adipose tissue, umbilical cord tissue and blood, amniotic membrane and dental pulp (Atluri et al., 2020). According to recent publications and by consulting the site www.clinicaltrials.gov, the umbilical cord is the most adequate source for MSCs, for several reasons: (i) high concentration of MSCs, (ii) UC-MSCs can be obtained during or after birth, by non-invasive techniques, (iii) the Wharton-jelly is considered a by-product, and several high quality samples are cryopreserved around the world in public and private high-regulated banks; (iv) UC-MSCs present faster doubling times, since these cells are closer to embryonic-stem-cells (ESCs) when compared to bone-marrow or adipose tissue MSCs; (v) on the other hand the UC-MSCs are safer than ESCs because are not tumorigenic; (vi) can be used as an allogenic treatment since these cells are immunoevasive and express low levels of major histocompatibility complex (MHC) class 1 molecules and no MHC class II (Caseiro et al., 2015, 2019). The intravenous administration is the selected route for the treatment with UC-MSCs, since most of the infused UC-MSCs will be trapped in the lungs, the most affected organ in COVID-19 patients (Wang et al., 2020).

As an important cell population, MSCs have the potential to conduct to very promising outcomes, when considered for the treatment of lung disease. The regulation of the immune system in the lung, by these cells, entails modulation of activation and effector function of immune cells, suppressing of infiltrated cells and diminishing edema (Liu et al., 2020). MSCs have been reported to efficiently cure ALI/ARDS, principally by paracrine mechanisms based on the action of EVs, such as microvesicles and exosomes (Chan et al., 2015). *In vitro* studies reported that UC-MSC demonstrated therapeutic efficacy on mice with ALI induced by influenza A (H5N1) virus. Researchers confirmed that UC-MSCs presented effective results for the restoration of damaged alveolar fluid clearance and protein permeability of influenza A infected alveolar epithelial cells (Fujita et al., 2018). Clinical studies made in patients with Influenza H7N9 virus, which presented symptoms such as ARDS, lung failure, and fulminant pneumonia, showed that MSCs are a promising choice for treating virus-induced pneumonia. Based on informed consent, 44 patients with H7N9 induced ARDS were included as the control group, while 17 patients with H7N9 induced ARDS served as the experimental group, injected with allogeneic menstrual blood-derived MSCs. The transplantation of MSCs significantly lowered the mortality rate compared with the control group, with no adverse effects. The results suggest MSCs improved the survival rate. Once Influenza A and COVID-19 share analogous symptoms (such as ARDS and lung failure) and similar multi-organ dysfunction, along with the positive results of *in vitro* tests and clinical studies, MSCs-based therapy are considered a promising therapeutic alternative for treating COVID-19 (Khatri et al., 2018).

### MSCs as a Treatment for COVID-19

To date, there is limited published information regarding COVID-19 disease and the potential efficacy of MSCs, although MSCs efficacy in influenza lung infections has been recently reported. MSCs can affect the performance of immune cells, both adaptive and innate, and therefore, MSCs therapy can theoretically inhibit the over-triggering of the immune system and promoting endogenous repair following SARS-CoV-2 infection. Several countries have begun clinical studies on MSCs therapies, and some positive results have been published. A study reported from China, on a 65-year-old female patient with a critical condition of COVID-19 presented promising outcomes after 21 days of treatment with UCB-MSCs. Initially, the patient showed neutrophil increase and lymphocyte decreased, and was treated with several antiviral drugs, while being subjected to non-invasive mechanical ventilation. As the vital signs deteriorated, the patient received 3 doses of single UCB-MSCs solution. The vital signs improved after the second MSCs administration as other symptoms gradually disappeared. Following, the patient was removed from the ventilator, with a normal number of immune system blood cells. The results suggested UCB-MSCs to be an alternative and effective treatment option for patients suffering with COVID-19, alone or in combination with other existing therapies (Chen et al., 2020). A different group in China of 7 patients with COVID-19 were selected, one with critical diagnosis, 4 with severe diagnosis, and 2 showing moderate symptoms. Three additional patients with severe diagnoses were chosen as placebo control. All patients presented pyrexia, breath impairment, and thus low oxygenation rates, and pneumonia. As the patients’ health status was aggravating, 1 million MSCs per kilogram body weight were administered. Patients were kept on observation during 2 weeks recovery period. Mitigation of the symptoms was observed 2–4 days after treatment, along with a decrease in pneumonia infiltration, with no apparent adverse effects. This study as the one previously described, demonstrated MSCs intravenous infusion as a safe and efficient therapeutic alternative for treating COVID-19 patients (Golchin et al., 2020). As mention before, as the immune system is over-triggered it leads to a cytokine storm. The cytokine storm is one of the worst consequences of the disease with the production of inflammatory cytokine accompanied by a weak interferon response. Notably, SARS-CoV-2 triggers pathogenic Th1 cells into secreting pro-inflammatory cytokines, as GM-CSF and interleukin-6 (IL-6). Furthermore GM-CSF activates CD14+CD16+ inflammatory monocytes into producing extensively IL-6, TNF-α. Thus, The COVID-19 consequent cytokine-storm constitutes on a large expression of IL-6 and TNF-α, but recent investigations also showed that high levels of IFN-y, interleukin (IL)- 1β, 2, 4, 6, 8, 10, 17, induced protein 10 (IP10), monocyte chemoattractant protein-1 (MCP-1), are also significantly elevated in patients with severe COVID-19 (Hu et al., 2020). Both studies presented before, included patients with those characteristics. However, the immunomodulation capacity of MSCs was the key to the promising results obtained and transplantation of MSCs also presented excellent outcomes. Suppressing over-triggering of the immune system and enhancing of the endogenous microenvironment repair capacity are some of the potential of MSCs therapies. Their particular immunomodulation potential results in reduced concentration levels in pro-inflammatory cytokines and chemokines in the patients’ serum. MSCs regulatory capacity also includes increased dendritic cells migrating to the injured tissue, as well as a minor migration of mononuclear/macrophages migration to the lesion site, resulting in a decrease of the levels of reactive C-protein (a biomarker for inflammation), a rise in oxygen saturation and improvement of lymphopenia (low level of lymphocytes in the blood). Moreover, the increase of IL-10 modulated the immune system and Vascular Endothelial Growth Factor (VEGF) also promoted angiogenesis conducting to lung repair. MSCs can release VEGF but also hepatocyte growth factor (HGF), which are able to stabilize endothelial barrier function by restoring pulmonary capillary permeability. By inhibiting pulmonary vascular endothelial cell apoptosis, enhancing the recovery of VE cadherin, and reducing pro-inflammatory factors, MSCs control inflammation and protect the lung endothelial barrier (Wang et al., 2020). In both studies intravenous infusion was applied, and it seems to be the most beneficial route of administration due to the fact that, some MSCs concentrate in the lung tissue, after intravenous infusion. Intuitively, this should likely be beneficial and enhance the pulmonary microenvironment recovery, protecting alveolar epithelial cells, preventing fibrotic tissue formation, thus improving pulmonary function (Bulut and Kato, 2020; Madabhavi et al., 2020). Those clinical trials also investigated if SARS-CoV-2 could infect the MSCs used, through the ACE-2 receptor widely distributed on human cells, but the MSCs that were initially negative, remained ACE-2 negative and free from COVID-19. The positive results of those two studies are thought to be related to immunomodulatory properties of MSCs as well as MSCs differentiation potential that can avoid lung tissue impairment, by blocking the cytokine storm and by promoting regenerative and reconstructive processes on the affected tissue. Furthermore, MSCs secretion of paracrine factors, and their interaction with immune cells, leads to immunomodulation and a robust anti-inflammatory activity (Chan et al., 2015).

### Future Challenges and Perspectives

Given the urgent need to find out an effective treatment, there are 3567 active clinical trials concerning COVID-19 at the date of October 1, 2020, involving several different approaches, several mechanisms of action, biomolecules and drugs. Knowing the potential of MSCs, and based on positive results already obtained, several clinical trials using MSCs have already began or are outlined to start shortly. Searching the words “COVID-19” and “Mesenchymal Stem Cells” is possible to identify 62 active trials. Of the 62 trials, 16 are located in the American continent, 9 are located in Europe, 11 in East Asia, and the other 26 are distributed over the remaining continents. Considering the different origins of MSCs, 17 of the clinical trials use UC-MSCs, 9 assays use AT-MSCs, 6 use BM-MCs, 5 use WJ-MSCs, 2 use DP-MSCs, 1 uses OM-MSCs, 1 uses MSCs derived exosomes, and 21 clinical trials do not specify concretely the source, or use more than one. Notably, 57 of the clinical trials are in Phase 1 or Phase 2, research phases to describe and collect preliminary data on the drug performance in people suffering with the disease, and 3 clinical trials are already in Phase 3, a phase of research that collects information on the pharmaceutical safety and effectiveness, analyzing diverse populations, dosages and application of the drug combined with others (research, in the ClinicalTrials.gov database, under the terms “MSCs,” on 10/1/2020).

## Conclusion

The current pandemic we are facing, that has not yet shown signs of weakening, is one of the great scourges of recent years and considering the need for mitigation, envisioning at keeping mortality rates low, there is an urgent need to discover effective mechanisms of prevention and therapies. Global efforts are focused on research and development of new therapies, testing different approaches, where MSCs based-therapies standing out. Regarding the pathogenesis of the SARS-CoV-2, several groups reported an initial recognition of the ACE-2 receptor. These are cell-membrane receptors, widely distributed, and particularly in alveolar cells. In addition to the mild symptoms as fever, cough, muscular soreness, expectoration, and dyspnea, COVID-19 can trigger an immune system overreaction, causing a cytokine storm followed by edema, inefficient gas exchange, ARDS, cardiac impairment, and secondary infection that can lead to death. The currently available cure of the COVID-19 disease is mainly dependent on the immune system function of the patient, therefore, avoiding cytokine overproduction may be decisive to recovery of SARS-CoV-2 patients. Current evidence suggests that therapies applying MSCs and MSCs secretome/EVs are a safe and an efficient approach for treating patients suffering from COVID-19 induced pneumonia, with the possibility to reverse severe critical disease with high potency, without the occurrence of severe adverse events. The immunomodulatory properties of MSCs can prevent lung tissue impairment by inhibiting pro-inflammatory cytokines overproduction and by promoting regeneration of the damaged tissue, as MSCs secrete a variety of paracrine factors, responsible for their immunomodulation and anti-inflammatory properties. Stem cell therapy, especially MSCs can potentially be an ideal therapy or be part of a combination of therapies to treat COVID-19 patients, nonetheless, since the number of patients who underwent this kind of treatment is still restricted, more studies with larger samples are needed to validate this therapeutic option, to comprehend the SARS-CoV-2 mechanism of action and to optimize this therapeutic option, by applying randomized studies promoting safety and efficacy of MSCs application on COVID-19 disease.

## Author Contributions

All authors listed have made a substantial, direct and intellectual contribution to the work, and approved it for publication.

## Conflict of Interest

The authors declare that the research was conducted in the absence of any commercial or financial relationships that could be construed as a potential conflict of interest.

## Abbreviations

AF: amniotic fluid
ALI: acute lung injury
ARSD: acute respiratory distress syndrome
AT: adipose tissue
AT-MSCs: adipose tissue mesenchymal stem cells
BM: bone marrow
BM-MSCs: bone marrow mesenchymal stem cells
CFU-F: colony forming unit-fibroblast
DP: dental pulp
DP-MSCs: dental pulp mesenchymal stem cells
EVs: extracellular vesicles
GM-CSF: granulocyte-macrophage colony-stimulating factor
HGF: hepatocyte growth factor
ISCT: The International Society for Cellular Therapy
MERS: middle east respiratory syndrome
MHC: major histocompatibility complex
MSCs: mesenchymal stem cells
OM-MSCs: olfactory mucosa mesenchymal stem/stromal cells
SARS: Severe Acute Respiratory Syndrome
TMPRSS2: transmembrane protease serine-2
UCB: umbilical cord blood
UCB-MSCs: umbilical cord blood mesenchymal stem cells
UCT: umbilical cord tissue
UC-MSCs: umbilical cord mesenchymal stem cells
WJ: Wharton’s Jelly
WJ-MSCs: Wharton’s Jelly mesenchymal stem cells.
